# Protective Effects of Chia Seeds and Vitamin E Against Benzo[a]Pyrene‐Induced Toxicity in Male Mice: A Comparison of co‐Administration and Pre‐Treatment Strategies

**DOI:** 10.1002/fsn3.72405

**Published:** 2026-10-07

**Authors:** Mohammad Rezaei, Maryam Karimi‐Dehkordi

**Affiliations:** ^1^ Animal and Animal Products‐Borne Diseases Research Center, IVO Tehran Iran; ^2^ Department of Food Hygiene, Faculty of Veterinary Medicine University of Tehran Tehran Iran; ^3^ Department of Veterinary, Shk. C Islamic Azad University Shahrekord Iran; ^4^ Research Center of Nutrition and Organic Products (R.C.N.O.P) Shahrekord Branch, Islamic Azad University Shahrekord Iran

**Keywords:** benzo[a]pyrene, chia seeds, food safety, liver toxicity, oxidative stress, vitamin E

## Abstract

Due to its carcinogenic and toxic effects, benzo[a]pyrene, which belongs to the family of polycyclic aromatic hydrocarbons, is a major health concern, primarily from sources such as tobacco smoke and grilling. This study investigated chia seeds and vitamin E as protective agents against benzo[a]pyrene toxicity in male mice. A total of 70 male mice were randomly assigned to 14 experimental groups (*n* = 5 per group): a negative control group, a vehicle control group (olive oil), a benzo[a]pyrene‐only group (20 mg/kg), a vitamin E‐only group (250 mg/kg), a vitamin E + benzo[a]pyrene co‐administration group, three chia seed‐only groups (10%, 20%, and 30% in diet), three chia seed + benzo[a]pyrene co‐administration groups, and three chia seed pre‐treatment groups (2 weeks of chia seeds alone followed by 4 weeks of chia seeds + benzo[a]pyrene). Biomarkers of oxidative stress, lipid profiles, and liver function were measured. Benzo[a]pyrene exposure significantly decreased superoxide dismutase and total antioxidant capacity, and altered lipid metabolism, increasing triglyceride (TG) and low‐density lipoprotein (LDL) levels. Interestingly, compared with those of the benzo[a]pyrene group, the total antioxidant capacity and superoxide dismutase activity of the chia seeds enriched with 20% and 30% benzo[a]pyrene notably increased, and the malondialdehyde level decreased. Moreover, the level of aspartate aminotransferase decreased. However, vitamin E had a minimal protective effect and did not reduce oxidative damage. In summary, chia seeds, unlike vitamin E, enhanced antioxidant defenses, improved liver function, and ameliorated lipid metabolism disturbances caused by benzo[a]pyrene. Chia seeds are a promising dietary option to counteract environmental toxicants such as benzo[a]pyrene.

## Graphical abstract

Benzo[a]pyrene (BaP), a polycyclic aromatic hydrocarbon found in tobacco smoke and grilled foods, induces DNA damage, oxidative stress, liver toxicity, and lipid metabolism disturbances. This study investigated the protective effects of chia seeds (10%, 20%, and 30% in the diet) and vitamin E (250 mg/kg) against BaP‐induced toxicity (20 mg/kg) in male mice. The study design included co‐treatment (4 weeks of simultaneous chia seeds and BaP) and pre‐treatment (2 weeks of chia seeds alone followed by 4 weeks of chia seeds + BaP). The key findings demonstrated that chia seeds at 20% and 30% significantly improved oxidative stress markers, including increased total antioxidant capacity (TAC) and superoxide dismutase (SOD) activity, and decreased malondialdehyde (MDA) levels. Chia seeds also improved liver function by reducing total bilirubin and AST levels and increasing albumin. Furthermore, chia seeds ameliorated lipid metabolism disturbances by reducing triglycerides and LDL and increasing HDL. The mechanism of BaP toxicity involves its metabolism by CYP1A1 and CYP1B1, overproduction of reactive oxygen species (ROS), lipid peroxidation, and subsequent liver injury. The protective role of chia seeds is attributed to their natural antioxidant and anti‐inflammatory properties, support for liver health, and improvement of lipid metabolism. In conclusion, chia seeds, unlike vitamin E, enhanced antioxidant defenses, improved liver function, and ameliorated lipid metabolism disturbances caused by BaP, making them a promising dietary option to counteract environmental toxicants such as BaP.

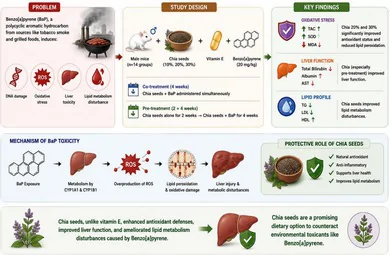

## 1 Introduction

Polycyclic aromatic hydrocarbons (PAHs) represent important environmental contaminants because they are toxic and bioaccumulate. Benzo[a]pyrene (BaP), a model PAH, is formed by the incomplete combustion of organic matter, including cigarette smoke, fossil fuels and grilled meats (Shariatifar et al. 2021). During the grilling of meats, fat burns down on the coal and smoke, which is rich in PAHs that fall into the food (Mariussen et al. 2024). BaP formation is dependent on the temperature, cooking time and type of fuel used, with high‐temperature and long‐duration grilling resulting in the highest level of contamination (Sholikin et al. 2025). Thus, it is classified as a Group 1 carcinogen by the International Agency for Research on Cancer (Das and Ravi 2022). In addition to exposure via the diet, occupational exposure to BaP and other PAHs still represents a considerable problem for coke oven workers in aluminum smelting plants, asphalt producers and coal gasification plants (Montero‐Pérez and Olivero‐Verbel 2024). Analyses have shown that the PAH concentration in some work areas, especially in the anode cooking and anode‐making zones of aluminum plants, represents a significant carcinogenic risk for workers, with BaP being the main constituent (Bahjati Ardakani et al. 2025). Workers in coke ovens have been identified as an occupationally high‐risk group for exposure to PAHs and require rapid implementation of preventative measures (Montero‐Pérez and Olivero‐Verbel 2024). Hence, immediate protective measures are necessary for public and occupational exposure. BaP is metabolically converted by cytochrome P450 enzymes, primarily CYP1A1 and CYP1B1, into activated intermediaries such as BaP‐7,8‐dihydrodiol‐9,10‐epoxide (BPDE), which form DNA adducts that cause carcinogenesis (Caipa Garcia et al. 2022). BaP metabolism also produces reactive oxygen species (ROS), which cause oxidative stress, lipid peroxidation, and cell dysfunction (Wang et al. 2024; Zhang et al. 2023). Since the liver is the detoxification organ of the body, it is highly sensitive to BaP‐induced hepatotoxicity (Bukowska and Duchnowicz 2022; Kim et al. 2024). The body's endogenous antioxidant defenses involve two parts: enzymes (glutathione peroxidase (GPx), superoxide dismutase (SOD), and catalase) and nonenzymatic antioxidants (vitamin E and reduced glutathione) (Bandodkar et al. 2025). Because the body's antioxidants can be overcome by the vast quantities of ROS generated upon exposure to BaP, a source of exogenous antioxidant supplement is needed (Mahdavi Yekta et al. 2020). *Salvia hispanica L*. (Chia seeds) are rich in omega‐3 fatty acids (ALAs), dietary fiber, and polyphenols, which confer anti‐inflammatory, antioxidant, and hepatoprotective properties (Marcinek and Krejpcio 2017; Oliveira‐Alves et al. 2017). These bioactive compounds are known to inhibit the damage caused by free radicals and increase internal antioxidant capacities. Chia seeds have been proposed as a part of dietary intervention against BaP toxicity (Sachi et al. 2024). Vitamin E is known to be a potent fat‐soluble antioxidant that prevents lipid peroxidation of membranes and stimulates internal antioxidant enzymes (Fatima et al. 2025). The present study investigated the beneficial effects of chia seeds and vitamin E on BaP‐induced toxicity in male mice by determining the following biomarkers in the serum: oxidative stress, liver function, renal function and lipid profile. The effect was studied under two intervention schemes: coadministration (chia seeds or vitamin E administered simultaneously with BaP for 4 weeks) and pre‐treatment (chia seeds administered for 2 weeks, then chia seeds + BaP administered for 4 weeks). This study is the first to simultaneously evaluate a natural whole‐food intervention (chia seeds) and a synthetic antioxidant (vitamin E), providing evidence for dietary advice for risk reduction associated with high exposure to BaP due to diet or work environment.

## 2 Materials and Methods

### 2.1 Ethical Approval and Animal Housing

All the experimental procedures involving animals were approved by the Institutional Animal Ethics Committee of Islamic Azad University, Shahrekord Branch, Iran (IR.IAU.SHK.REC.2024.035), and all the experiments were performed according to the ARRIVE guidelines. Seventy male C57BL/6 mice (6 weeks of age, weighing 15–20 g) were housed in individually ventilated cages for 7 days to habituate to the environmental conditions at a controlled temperature (25°C ± 1°C) and humidity (55% ± 5%) under a 12‐h light/dark cycle. The mice were fed normal laboratory food and sterile water ad libitum. The mice were housed in standard cages enriched with PVC tunnels, wooden blocks for gnawing and paper for nesting. These materials were included to reduce the monotony of the environment, as well as social stress during the course of the experiment (Kilkenny et al. 2010). The experimental group assignment for the mice is described in the Study Design section. All histopathology and biochemistry data were analyzed in a blinded manner so that the researchers did not know the treatment group of any sample until after the analyses were complete. Mice were assessed for humane endpoints (e.g., > 20% body weight loss, lethargy) on a daily basis. None of the mice met these criteria during the course of the 4‐week study.

### 2.2 Chemicals and Reagents

All the chemicals and reagents used were of analytical grade (≥ 99% purity, Merck, Darmstadt, Germany), and double distilled water was used for dilution in all the cases. The source of BaP was Sigma–Aldrich, with a purity of 96%. The chemical mixture was handled under low light conditions to minimize the risk of chemical degradation.

To provide stability, chemical BaP and vitamin E were dissolved in pharmaceutically graded olive oil, light‐proof with amber glass vials and stored at 40°C. Olive oil was selected as the vehicle because it is a standard inert solvent for lipophilic compounds in oral gavage studies and does not interfere with oxidative stress or biochemical parameters (Ikeda et al. 2024). Fresh working solutions were prepared on a weekly basis to circumvent any possible photodegradation and to ensure that an unchanging toxic dose was delivered throughout the 4‐week study.

### 2.3 Preparation of Black Chia Seeds

The black chia seeds were provided by a local commercial supplier (Tehran, Iran). The seeds were then powdered and mixed directly with standard rodent food at concentrations of 10%, 20%, and 30% (w/w). All the groups were provided with a diet, and food consumption was checked daily.

### 2.4 Study Design

The sample was composed of 70 male mice divided randomly among 14 groups (*n* = 5 per group) through the use of a computerized random number generator. The sample size was determined via power analysis via G*Power software (version 3.1.9.7). With one‐way analysis of variance, a large expected effect size (f = 0.40) at an alpha level of 0.05 and a required statistical power level of 0.80, a total sample size of at least 70 was set to guarantee statistical power for detecting meaningful differences, promoting adherence to 3 Rs (reduction) and limiting the wasteful usage of animals. A dose of 20 mg/kg BaP was chosen because previous studies demonstrated that this dose induces significant oxidative stress (elevated MDA, reduced SOD and GPx), hepatorenal injury, and reproductive toxicity in murine models, while remaining below the acute lethal threshold (Luderer et al. 2019; Rahmani et al. 2021). For the reference antioxidant standard, a vitamin E dose of 250 mg/kg was used as the reference antioxidant, as this dose has been shown to exert significant antigenotoxic and antioxidant effects against BaP‐induced damage in murine models (Slapšytė et al. 2011). Three doses of chia seeds (10, 20 and 30 w/w in the diet) were tested. The 10% and 20% doses were selected based on previous reports of antioxidant and hepatoprotective effects in rodent models (da Silva Marineli et al. 2015), and the 30% dose was included to evaluate dose‐dependent protective responses. A 14‐group design was used so that each dose could be compared under two distinct sets of conditions: pre‐treatment model to assess the prevention capability of the treatment and co‐administration model to assess whether damage, once it has already been incurred, could be reduced. Each dose was given by oral gavage once daily between 8 a.m. and 10 a.m. daily for 4 weeks, except when specifically stated (Zhakipbekov et al. 2024).

### 2.5 Group Allocation

The animals were divided into the following categories:

- Group 1: Negative control (received a normal diet).
- Group 2: vehicle control group (0.04 mL of olive oil was given by gavage daily for 4 weeks) (Ikeda et al. 2024).
- Group 3: BaP group (which received 20 mg/kg BaP powder dissolved in olive oil (as the vehicle) by gavage for 4 weeks; for an ~25 g mouse, the concentration was 0.5 mg of BaP per day) (Luderer et al. 2019; Rahmani et al. 2021).
- Group 4: Vitamin E group (250 mg/kg vitamin E powder dissolved in olive oil by gavage daily for 4 weeks; for an ~25 g mouse, this value was 6.25 mg of vitamin E per day) (Slapšytė et al. 2011).
- Group 5 (Co‐administration): Vitamin E + BaP group received 250 mg/kg vitamin E powder dissolved in olive oil together with 20 mg/kg BaP in olive oil by gavage daily for 4 weeks.
- Groups 6–8: Chia‐only groups (received 10%, 20%, or 30% chia seeds mixed in food for 4 weeks) (da Silva Marineli et al. 2015).
- Groups 9–11 (Co‐administration): Chia + BaP groups (received 10%, 20%, or 30% chia seeds mixed in food together with 20 mg/kg BaP by gavage daily for 4 weeks).
- Groups 12–14 (Pre‐treatment): Pre‐treatment Chia + BaP groups (chia seeds alone were mixed in food for the first 2 weeks, followed by chia seeds together with 20 mg/kg BaP by gavage for the subsequent 4 weeks).

### 2.6 Serum Collection and Processing

The mice were fasted overnight (12 h) and then anesthetized with ketamine and xylazine (ketamine 90 mg/kg and xylazine 4 mg/kg). Cardiac puncture blood was collected in plain tubes and centrifuged at 3000 × g for 15 min. Serum was then stored at −80°C until analysis (Underwood and Raymond 2020).

### 2.7 Serum Biochemical Analysis

The following serum parameters were assessed: (i) oxidative stress markers (total antioxidant capacity (TAC), SOD, GPx, and malondialdehyde (MDA)); (ii) liver function parameters (total bilirubin, albumin (Alb), fasting blood sugar (FBS), alanine aminotransferase (ALT), aspartate aminotransferase (AST), and alkaline phosphatase (ALP)); (iii) renal function parameters (blood urea nitrogen (BUN) and creatinine (Crt)); and (iv) lipid profiles (TG, total cholesterol (TC), LDL (low‐density lipoprotein), and high‐density lipoprotein (HDL)).

TAC was determined in serum via the ferric reducing ability of plasma (FRAP) assay (Karimi‐Dehkordi et al. 2025), a technique that has been modified to quantify oxidative stress parameters as described in other reproductive and biochemical studies (Benzie and Devaki 2018). Essentially, the assay measures the decrease in absorbance at 593 nm, as ferric‐tripyridyltriazine (Fe3 + ‐TPTZ) is reduced to its ferrous form (Fe2+). The activities of SOD were determined by the autoxidation of pyrogallol (Mohammadi et al. 2024) and measured throughout the experiment in terms of U/mg protein (units of activity). Superoxide dismutase (SOD) activity was measured as the amount of enzyme that was capable of reducing the rate of oxidation of pyrogallol by 50%. GPx activity was measured spectrophotometrically by the rate of oxidation of NADPH at 340 nm. The method was based on the same established enzymatic methods used previously to assess oxidative stress during reproductive studies (Izanloo et al. 2022; Mohammadi et al. 2024). Briefly, GPx catalyzes the oxidation of glutathione by cumene hydroperoxide, and subsequently, in the presence of glutathione reductase and NADPH, oxidized glutathione is immediately restored to its reduced form with simultaneous oxidation of NADPH to NADP+. MDA levels were determined via the Thiobarbituric Acid Reactive Substances (TBARS) assay (Izanloo et al. 2021; Karimi‐Dehkordi et al. 2024).

Biochemical markers such as Alb, FBS, total bilirubin, TG, TC, HDL, and LDL were measured with Ziest Chem Diagnostic animal biochemistry kits (Tehran, Iran). ALT, ALP, AST, BUN, and Crt levels were assessed with diagnostic kits from Pars Azmoon (Tehran, Iran). All bioassays were conducted according to the instructions of the respective manufacturers (Karimi‐Dehkordi et al. 2025).

### 2.8 Statistical Analysis

Statistical analyses were performed via SPSS software, version 24. The normal distribution of the data was verified via the Shapiro–Wilk test (all groups *p* > 0.05). Homogeneity of variances was confirmed with Levene's test (all parameters *p* > 0.05). One‐way ANOVA was used to analyze mean differences among groups, followed by Tukey's post hoc test to distinguish specific group comparisons. All values are reported as the means ± SEMs. Statistical significance was set at a *p* value < 0.05.

## 3 Results

### 3.1 Oxidative Stress Parameters

As shown in Table 1, TAC and SOD activity were significantly lower in the BaP group than in the control group (*p* < 0.05). Neither of these effects was ameliorated by vitamin E. Compared with BaP, 20% and 30% chia seed co‐administration significantly elevated TAC and SOD activities and reduced MDA activity. SOD and GPx activities increased, and the MDA level decreased with 30% chia seed pretreatment.

**TABLE 1:** Evaluation of oxidative stress indicators among different groups.

Groups	TAC (μmol/L)	SOD (U/mL)	GPx (U/mL)	MDA (μmol/L)
Control	6.38 ± 0.455^fgh^	63.1 ± 1.194^cd^	54.98 ± 0.928^def^	5.6 ± 0.36^bcde^
Vehicle control	6.4 ± 0.43^fgh^	62.2 ± 0.908^cd^	54.52 ± 1.043^def^	5.72 ± 0.258^bcde^
BaP	4.62 ± 0.61^abc^	53.2 ± 3.7^a^	50.08 ± 0.73^bcd^	6.76 ± 0.195^de^
Vit E	7.26 ± 0.503^h^	65.46 ± 0.456^d^	58.2 ± 1.304^f^	5.04 ± 0.23^bcde^
Vit E + BaP	5.22 ± 0.19^bcde^	54.04 ± 3.7^ab^	51.66 ± 0.654^cde^	6.8 ± 0.158^e^
Chia‐only				
Chia 10%	6.04 ± 0.182^efg^	62.72 ± 0.835^cd^	53.82 ± 2.883^def^	4.22 ± 0.67^abc^
Chia 20%	6.22 ± 0.23^efgh^	62.9 ± 1.432^cd^	55.22 ± 0.823^def^	3.82 ± 0.683^abc^
Chia 30%	6.6 ± 0.1^gh^	64.56 ± 1.53^d^	56.96 ± 3.522^ef^	2.2 ± 0.57^a^
Co‐administration				
Chia 10% + BaP	4.92 ± 0.277^abcd^	54.4 ± 5.46^ab^	41.8 ± 2.5^a^	6.9 ± 1.876^e^
Chia 20% + BaP	5.36 ± 0.25^cdef^	59.66 ± 0.44^bcd^	45.4 ± 3.507^ab^	6.14 ± 2.023^cde^
Chia 30% + BaP	5.736 ± 0.621^defg^	62.14 ± 1.299^cd^	45.8 ± 3.767^abc^	5.34 ± 2.36^bcde^
Pre‐treatment				
Chia 10% + BaP	4.28 ± 0.646^ab^	56 ± 4.899^ab^	52.2 ± 1.923^de^	4.44 ± 0.83^abcd^
Chia 20% + BaP	4.04 ± 0.365^a^	57.92 ± 2.64^abc^	52.98 ± 1.418^def^	4.34 ± 0.371^abc^
Chia 30% + BaP	4.92 ± 1^abcd^	63.2 ± 1.304^cd^	57.5 ± 5.895^ef^	3.7 ± 0.245^ab^

*Note:* The presence of at least one common superscript letter in each column indicates no statistically significant difference between the corresponding groups (*p* > 0.05).

Abbreviations: BaP, Benzo[a]pyrene 20 mg/kg; GPx, Glutathione peroxidase; MDA, Malondialdehyde; SOD, Superoxide dismutase; TAC, Total antioxidant capacity.

### 3.2 Liver Function Parameters

As shown in Table 2, BaP dose dependently increased AST (*p* < 0.05). Vitamin E had no protective influence. Co‐administration of chia seeds (chia seed protection) decreased AST at all doses and rescued the Alb at 10% and 20%. Sensitization to 30% chia seeds significantly decreased the FBS and AST contents.

**TABLE 2:** Evaluation of hepatic function parameters among different groups.

Groups	Total bill (mg/dL)	Alb (mg/dL)	FBS (mg/dL)	ALT (IU/L)	AST (IU/L)	ALP (IU/L)
Control	0.82 ± 0.286^ab^	4.3 ± 0.583^cde^	92.2 ± 4.5^abc^	44.6 ± 1.516	44.4 ± 1.67^ab^	51 ± 0.707
Vehicle control	0.9 ± 0.1^abc^	4.02 ± 0.593^cde^	93 ± 4.123^abc^	45.4 ± 1.673	44 ± 2.54^ab^	53.6 ± 0.894
BaP	1.592 ± 0.223^bcd^	2.78 ± 0.277^bc^	107.8 ± 25.104^c^	54.2 ± 3.701	62.2 ± 4.32^d^	59 ± 3.391
Vit E	1 ± 0.212^abc^	4.12 ± 0.192^cde^	89.8 ± 4.38^abc^	45.94 ± 1.521	48.94 ± 1.47^abc^	52.52 ± 1.244
Vit E + BaP	1.34 ± 0.241^abcd^	3.762 ± 0.415^cde^	99.8 ± 12.93^bc^	47 ± 2.915	54.78 ± 1.15^bcd^	55.38 ± 5.56
Chia‐Only						
Chia 10%	0.76 ± 0.297^a^	4.5 ± 1.326^de^	91.6 ± 6.877^abc^	46.2 ± 13.03	40.4 ± 4.39^a^	47.2 ± 5.12
Chia 20%	0.84 ± 0.358^ab^	2.88 ± 0.82^cd^	79.4 ± 6.23ab	47.8 ± 4.438	40.2 ± 7.12^a^	48 ± 6.964
Chia 30%	1.036 ± 0.613^abc^	4.36 ± 0.904^cde^	74.8 ± 2.68^a^	52.2 ± 13.498	39.4 ± 8.70^a^	47.2 ± 8.44
Co‐administration						
Chia 10% + BaP	2 ± 0.255^d^	4.76 ± 0.62^e^	94.6 ± 6.841^abc^	56.6 ± 12.178	44.8 ± 2.94^ab^	63.2 ± 11.009
Chia 20% + BaP	1.68 ± 0.567^cd^	4.84 ± 0.643^e^	86 ± 10.025^ab^	52.8 ± 19.46	46.40 ± 5.81^ab^	61 ± 22.17
Chia 30% + BaP	1.4 ± 0.453^abcd^	3.08 ± 1.462^cd^	82.8 ± 6.38^ab^	53.6 ± 15.176	43.40 ± 1.67^ab^	61 ± 26.058
Pre‐treatment						
Chia 10% + BaP	1.14 ± 0.23^abc^	0.426 ± 0.248^a^	96 ± 2.74^bc^	60 ± 20.676	51.20 ± 2.58^abcd^	45.4 ± 11.194
Chia 20% + BaP	1.22 ± 0.295^abcd^	0.66 ± 0.416^a^	92.8 ± 2.863^abc^	50 ± 5.83	60.20 ± 6.05^cd^	60.4 ± 14.258
Chia 30% + BaP	1.3 ± 0.51^abcd^	1.198 ± 0.319^ab^	84.8 ± 6.76^ab^	56.8 ± 20.85	49.60 ± 11.63^abc^	44 ± 6.041

*Note:* The presence of at least one common superscript letter in each column indicates no statistically significant difference between the corresponding groups (*p* > 0.05).

Abbreviations: Alb, Albumin; ALP, Alkaline Phosphatase; ALT, Alanine Aminotransferase; AST, Aspartate Aminotransferase; BaP, Benzo[a]pyrene 20 mg/kg; FBS, Fasting Blood Sugar; Total Bill, Total Bilirubin.

### 3.3 Renal Function Parameters

As shown in Figure 1, BaP increased the creatinine and BUN levels (*p* < 0.05). No intervention significantly enhanced these parameters.

**FIGURE 1:**
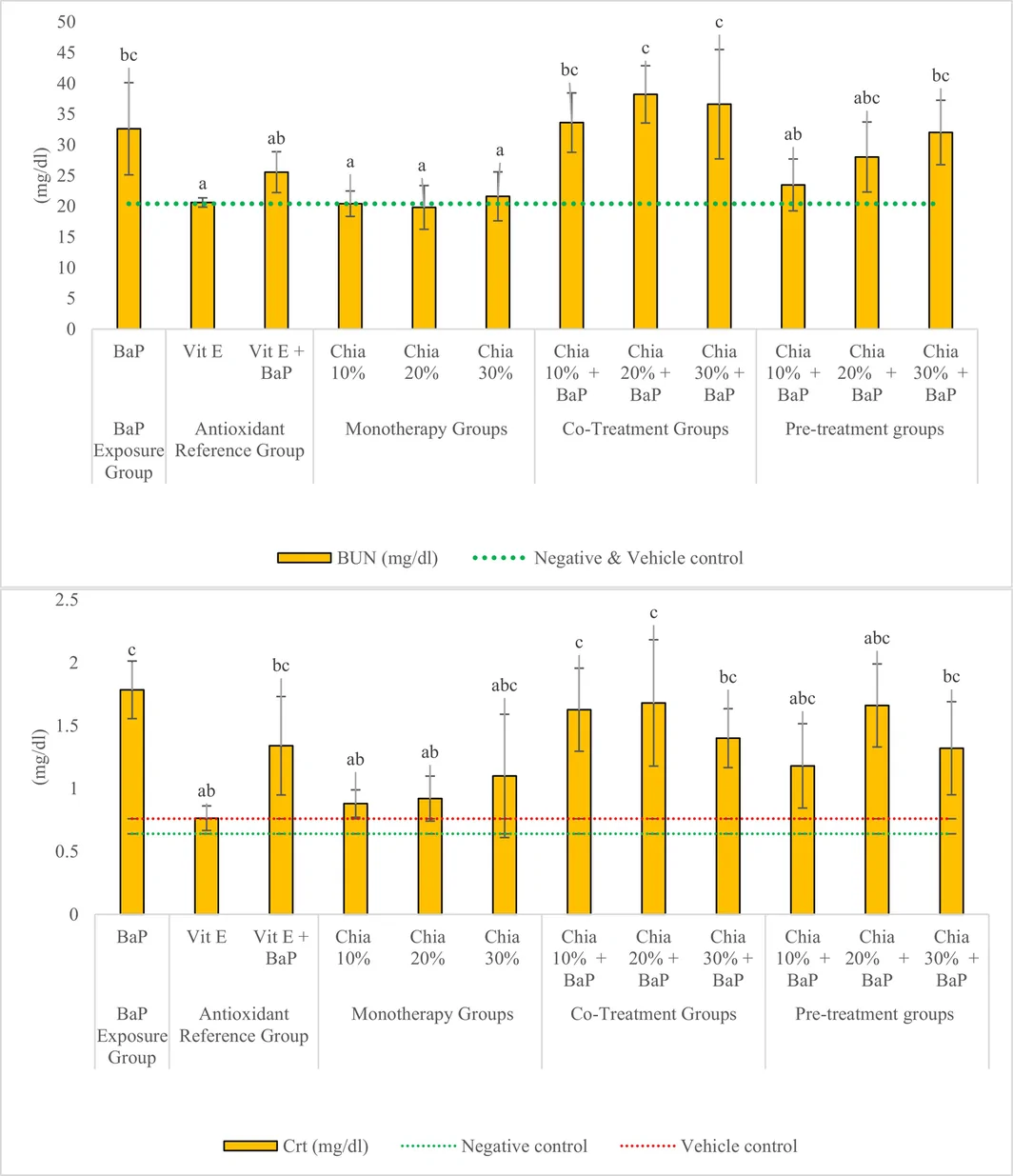
Evaluation of renal function parameters among different groups. The presence of at least one common superscript letter among the compared groups indicates no statistically significant difference (*p* > 0.05).

### 3.4 Lipid Profile

Table 3 shows that BaP significantly elevated TG and LDL levels (*p* < 0.05). Vitamin E Co‐administration reduced TG and TC levels. Chia seeds (Co‐administration) lowered TG at all doses and elevated HDL at 20% and 30% doses. Prior treatment with chia seeds decreased TG at all doses and increased HDL at the 30% dose. Olive oil (vehicle control) had no significant effect on any parameter compared with the control group.

**TABLE 3:** Evaluation of lipid profile parameters among different groups.

Groups	TG (mM)	TC (mM)	LDL (mg/dL)	HDL (mg/dL)
Control	1.10 ± 0.15^abc^	2.46 ± 1.31^abc^	82.60 ± 20.02^a^	65.00 ± 7.20^a^
Vehicle control	1.06 ± 0.23^ab^	2.86 ± 0.55^abcd^	80.80 ± 17.54^a^	65.80 ± 5.12^a^
BaP	2.44 ± 0.25^e^	4.60 ± 1.23^cd^	162.80 ± 44.20^b^	93.80 ± 15.38^abc^
Vit E	0.72 ± 0.17^a^	1.72 ± 0.85^a^	71.00 ± 14.61^a^	64.80 ± 6.30^a^
Vit E + BaP	1.76 ± 0.503^d^	2.02 ± 0.94^ab^	99.20 ± 10.57^ab^	78.80 ± 6.61^ab^
Chia‐Only				
Chia 10%	1.02 ± 0.13^ab^	3.02 ± 1.05^abcd^	95.40 ± 31.89^ab^	79.80 ± 12.21^ab^
Chia 20%	1.14 ± 0.24^abc^	2.68 ± 1.00^abcd^	88.80 ± 52.39^ab^	74.40 ± 16.70^ab^
Chia 30%	0.76 ± 0.11^a^	2.74 ± 0.90^abcd^	87.20 ± 33.77^ab^	132.00 ± 17.60^cde^
Co‐administration				
Chia 10% + BaP	1.38 ± 0.19^bcd^	5.04 ± 0.43^d^	119.40 ± 14.11^ab^	136.60 ± 30.68^cde^
Chia 20% + BaP	1.44 ± 0.25^bcd^	4.98 ± 0.96^d^	126.60 ± 39.03^ab^	150.60 ± 31.63^de^
Chia 30% + BaP	1.16 ± 0.24^abc^	4.24 ± 0.84^bcd^	120.80 ± 29.98^ab^	162.40 ± 20.40^e^
Pre‐treatment				
Chia 10% + BaP	1.82 ± 0.40^d^	4.74 ± 1.61^cd^	83.80 ± 40.64^a^	115.00 ± 19.77^bcd^
Chia 20% + BaP	1.68 ± 0.37^cd^	4.66 ± 1.03^cd^	124.40 ± 34.22^ab^	98.40 ± 20.50^abc^
Chia 30% + BaP	1.10 ± 0.12^abc^	4.64 ± 1.70^cd^	98.40 ± 64.04^ab^	145.40 ± 35.35^de^

*Note:* The presence of at least one common superscript letter in each column indicates no statistically significant difference between the corresponding groups (*p* > 0.05).

Abbreviations: BaP, Benzo[a]pyrene 20 mg/kg; HDL, High‐Density Lipoprotein; LDL, Low‐Density Lipoprotein; TC, Total Cholesterol; TG, Triglyceride.

## 4 Discussion

This study was designed to compare the capacity of chia seeds and vitamin E to exert protective effects on BaP‐administered male mice. Previous studies have examined one specific antioxidant.

However, this study examined the effects of the whole food matrix, where three key bioactive compounds, namely, chia seeds, α‐linolenic acid (omega‐3), dietary fiber, and polyphenols, may act synergistically to provide superior protection to a single compound against BaP‐induced oxidative stress and organ toxicity.

Various studies have confirmed the antioxidant and hepatoprotective activities and effects of natural compounds. Similar to our results obtained using chia seeds, other peptide‐based intervention studies have reported that these interventions reduce the effects of food‐borne toxins (Mahdavi‐Yekta et al. 2024). BaP is a model polycyclic aromatic hydrocarbon that is an environmental carcinogen produced by the incomplete combustion of organic matter, such as fossil fuels, smoking, or barbecued foods (Adeyeye and Ashaolu 2022). Once metabolically activated by CYP1A1 and CYP1B1, BaP was shown to act via reactive intermediate metabolites such as BPDE to generate pathways for DNA adduct formation, oxidative stress, and cell damage (Caipa Garcia et al. 2022). In the case of BaP, CYP1A1 was shown to be involved in the detoxification of oral BaP, whereas CYP1B1 facilitated the formation of more toxic metabolites, providing the basis for the effects on oxidative stress and tissue damage observed in this study.

In line with prior studies (Meng et al. 2021; Wang et al. 2023), pyrene treatment resulted in a significant reduction in total antioxidant capacity and superoxide dismutase activity and increased MDA levels. A decrease in superoxide dismutase results in a reduction in antioxidant defense against superoxide radicals, and an increase in MDA is related to damage to membrane lipids (Pizzino et al. 2017).

BaP exposure also leads to significant increases in aspartate aminotransferase, alanine aminotransferase, and alkaline phosphatase levels and decreases in the concentration of Alb, indicating hepatocellular damage and failure (Abomosallam et al. 2025; Karimpour Malekshah et al. 2022; Montero‐Pérez and Olivero‐Verbel 2024). Triglyceride and low‐density lipoprotein levels increased, which verified the disturbance of lipid metabolism by BaP (Bu et al. 2024). The creatinine and urea levels are increased, resulting in renal damage (Khattab et al. 2021; Ge et al. 2022).

Chia seed supplementation of 20% and 30% significantly increased GPx and total antioxidant capacity and decreased MDA concentrations to exhibit antioxidant protective effects, likely related to the high levels of alpha‐linolenic acid/omega‐3 fatty acids/polyphenols contained in chia seeds (Tavera‐Hernández et al. 2023). Chia seed supplementation restored the elevated aspartate aminotransferase concentrations to normal, increased the Alb concentration, and decreased TG and LDL while increasing high‐density lipoprotein levels, indicative of a protective effect comparable to that observed in Poudyal et al. (2012) (Poudyal et al. 2012).

The mechanisms underlying the lipid‐lowering effects of chia could involve both mechanistic pathways. Both the activation of peroxisome proliferator‐activated receptor alpha and the suppression of sterol regulatory element‐binding protein‐1 by chia‐derived ALA may occur simultaneously, resulting in an increase in fatty acid oxidation and a reduction in lipogenesis, leading to decreased levels of fatty acid synthase and acetyl‐CoA carboxylase activity and increased levels of activity of oxidative enzymes such as carnitine palmitoyltransferase‐1 and fatty acid oxidation enzymes (Rossi et al. 2013). In addition, chia seeds inhibit fatty acid translocase (CD36) expression, thereby inhibiting the uptake of fatty acids into tissues (Creus et al. 2016). Chia proteins and polyphenols also modulate inflammatory and lipid‐regulating pathways by reducing the levels of tumor necrosis factor alpha and sterol regulatory element‐binding protein‐1 while increasing adiponectin levels. Finally, polyphenols are inducers of pancreatic lipase activity and inhibit the oxidation of LDL. Overall, these mechanisms may account for the improved lipid profile found in this study. The addition of vitamin E (250 mg/kg) from BaP improved oxidative stress and hepatotoxicity to some extent, but much greater benefits were obtained from chia seeds. While vitamin E is known to be a strong free radical scavenger and lipid peroxidation inhibitor (Zingg 2015), the low effectiveness of vitamin E in this study may be due to the dosage given, the duration of treatment or the degree of oxidative damage caused by BaP. The current findings are similar to observations that vitamin E is less effective than other antioxidant combinations are (Gultekin et al. 2001; Kim et al. 2024). Research has indicated that vitamin E might influence CYP1A1 and CYP1B1 functions to improve detoxification and decrease bioactivation of BaP. Nonetheless, the results of this study suggest that this mechanism does not account for the complicated and multifaceted way in which BaP is toxic (Zhu et al. 2014). The outstanding protective activities of chia seeds are likely attributable to their multitarget, multimechanistic characteristics. Chia seeds are a significant source of alpha‐linolenic acid, omega‐3, and polyphenol‐rich s are (Gultekin et al. 2001; Kim et al. 2024). Research has indicated that vitamin E might influence CYP1A1 and CYP1B1 functions to improve detoxification and decrease bioactivation of BaP. Nonetheless, the results of this study suggest that this mechanism does not account for the complicated and multifaceted way in which BaP is toxic (Zhu et al. 2014). The outstanding protective activities of chia seeds are likely attributable to their multitarget, multimechanistic characteristics. Chia seeds are a significant source of alpha‐linolenic acid, omega‐3, and polyphenol‐rich foods, which have synergistic antioxidant effects greater than those of any other single agent, such as vitamin E (De Falco et al. 2018). In addition, chia components have been reported to induce the activation of endogenous antioxidant defense systems, such as the nuclear factor erythroid 2‐related factor 2 (Nrf2) signaling pathway, which can increase antioxidant gene expression (Joubert et al. 2022). Additionally, they can directly reduce ROS and avoid oxidative damage to lipids, proteins, and DNA. The dose of vitamin E provided, although it has already been reported to be sufficient, may be insufficient to suppress the activity of CYP1B1 or to protect against the massive oxidation stress of BaP in this model.

Overall, the greater benefit of chia seeds than vitamin E may be attributed to both greater antioxidant power and the combined effects of multiple pathways implicated in oxidative stress, inflammation, and metabolic dysfunction. This may account for the greater protection afforded against BaP‐induced toxicity. Interestingly, no renal biomarkers were significantly altered following any of the treatments, indicating that renal protection may require prolonged treatment times or the application of different therapies (Barangi et al. 2023). In line with these findings, a similar attenuated benefit of diet antioxidants on environmentally mediated nephrotoxicity has been observed in studies on *Lavandula officinalis* extract in a lead‐induced model (Karimi‐Dehkordi et al. 2025).

Another study revealed that pomegranate peel powder is a natural antioxidant‐rich byproduct that can ameliorate cadmium intoxication in Japanese quail. This could indicate the effectiveness of the plant extracts against heavy metal nephrotoxicity (Karimi‐Dehkordi et al. 2024). In conclusion, these results suggest that the effects of antioxidants in the kidney may be more restrictive than those in other organs and that specific studies *are needed. These results suggest that*.

Notably, several human studies have reported minor changes in lipid profiles and inflammatory markers among healthy/overweight individuals following chia supplementation (Nieman et al. 2009; Parker et al. 2018; Teoh SiewLi et al. 2018). Factors such as dose, duration of the study, subject characteristics, and lack of specific toxic challenges could be the reasons behind the inconsistency. Herein, we provide evidence of the beneficial effects of chia seeds under particular conditions of oxidative stress induced by BaP.

### 4.1 Limitations of the Study

This study did not employ an isocaloric diet, nor did it monitor body weight or food consumption. Additionally, tissue‐level analyses and effect sizes were not included. Furthermore, the combined effect of vitamin E and chia seeds was not evaluated in this study. This approach could reduce the required dose of chia seeds and thereby minimize their associated side effects while maintaining their protective efficacy. This remains an important direction for future research. These factors should be addressed in future research.

## 5 Conclusion

In this study, chia seeds might be a nutritional approach to alleviate the damaging effects of BaP. The fact that chia seeds are better than vitamin E is an indication that a whole food product with diverse bioactive compounds may have a stronger ability to protect against environmental toxicity‐induced damage than individual antioxidants do. A novel aspect of this work is that it offers support for whole‐food‐based diet therapies through direct comparison of a functional food and a typical antioxidant. Additionally, the effectiveness of pre‐treatment over simultaneous treatment indicates that preventive nutritional supplementation is crucial in preventing toxicant‐mediated damage. Because most people are exposed to BaP via their diet and environment, nutritional preventative measures to protect public health could be provided. Nevertheless, more clinical and mechanistic studies are needed to provide convincing evidence of such effects in humans and explain the biological mechanisms involved.

## Author Contributions

**Mohammad Rezaei:** funding acquisition, resources, writing – review and editing, writing – original draft, validation, methodology, data curation. **Maryam Karimi‐Dehkordi:** conceptualization, investigation, visualization, software, formal analysis, project administration, supervision.

## Funding

The authors have nothing to report.

## Conflicts of Interest

The authors declare no conflicts of interest.

## Data Availability

The data that support the findings of this study are available on request from the corresponding author. The data are not publicly available due to privacy or ethical restrictions.
